# The effect of chlormadinone acetate on odontogenic differentiation of human dental pulp cells: in vitro study

**DOI:** 10.1186/s12903-017-0379-0

**Published:** 2017-05-26

**Authors:** Se-Min Kim, Bin-Na Lee, Jeong-Tae Koh, Hoon-Sang Chang, In-Nam Hwang, Won-Mann Oh, Kyung-San Min, Yun-Chan Hwang

**Affiliations:** 1 Department of Conservative Dentistry, School of Dentistry, Dental Science Research Institute, Chonnam National University, Youngbong-ro 77, Buk-gu, Gwangju, 61186 Korea; 2 Department of Pharmacology and Dental Therapeutics, School of Dentistry, Dental Science Research Institute, Chonnam National University, Youngbong-ro 77, Buk-gu, Gwangju, 61186 Korea; 3 Research Center for Biomineralization Disorders, Chonnam National University, Youngbong-ro 77, Buk-gu, Gwangju, 61186 Korea; 4 Department of Conservative Dentistry, School of Dentistry, Chonbuk National University, 567 Baekje-daero, Deokjin-gu, Jeonju-si, 54896 Korea; 5 Research Institute of Clinical Medicine of Chonbuk National University-Biomedical Research Institute of Chonbuk National University Hospital, 20 Geonji-ro, Deokjin-gu, Jeonju-si, 54907 Korea

**Keywords:** Chlormadinoe acetate, Differentiation, Progesterone, Odontoblast, Dentin sialophosphoprotein, Dentin matrix protein-1, Dental pulp cell

## Abstract

**Background:**

Chlormadinone acetate (CMA) is a derivative of progesterone and is used as an oral contraceptive. The aim of this study was to investigate the effects of CMA on odontogenic differentiation and mineralization of human dental pulp cells (hDPCs) and related signaling pathways.

**Methods:**

Cell viability was determined by the water-soluble tetrazolium (WST)-1 assay. Odontogenic differentiation of hDPCs was evaluated by real-time polymerase chain reaction using odontogenic marker genes, such as alkaline phosphatase (ALP), osteocalcin (OCN), dentin sialophosphoprotein (DSPP), and dentin matrix protein-1 (DMP-1). Mineralization of hDPCs was evaluated by ALP staining and alizarin red staining. The extracellular signal-regulated kinase (ERK) pathway was examined by Western blot analysis.

**Results:**

There was no statistically significant difference in cell viability between the control and CMA-treated groups. Our analysis of odontogenic marker genes indicated that CMA enhanced the expression of those genes. CMA-treated hDPCs showed increased ALP activity and formation of mineralized nodules, compared with control-treated cells. In addition, CMA stimulation resulted in phosphorylation of ERK and resulted in inhibition of downstream molecules by the ERK inhibitor U0126.

**Conclusions:**

These findings suggest that CMA improves odontogenic differentiation and mineralization of hDPCs through the ERK signaling pathway.

## Background

Dental pulp is made up of loose connective tissue. However, in comparison to other types of connective tissue, dental pulp has several unique properties, such as the presence of odontoblasts, absence of histamine-releasing mast cells, tissue confinement in a hard cavity with little collateral circulation, and vascular access that is limited to the root apex [1]. Dental pulp is often subject to damage or injury, and, in most cases, dental pulp cells can differentiate to odontoblast-like cells, secreting a reparative or tertiary dentin [2, 3]. This suggests that the dentin–pulp complex has regenerative potential in response to injury.

Dental tissue has functional, developmental, and anatomical similarities to bone. The maintenance of hard tissue in the tooth or bone depends upon the stimulation of morphologically and functionally related cells, odontoblasts or osteoblasts, which are derived from the same mesenchymal stem cells [4]. Odontoblasts and osteoblasts secrete the same collagenous and non-collagenous proteins, such as osteonectin, osteocalcin (OCN), osteopontin, and bone sialoprotein [5]. Dentin matrix protein-1 (DMP-1) and dentin sialophosphoprotein (DSPP) were originally considered dentin-specific non-collagenous proteins [6], but several studies have revealed that DMP-1 and DSPP are also expressed in bones [7]. Therefore, these proteins have been used as mineralization markers for the odontogenic or osteogenic differentiation of human dental pulp cells (hDPCs).

Many studies have demonstrated that hard tissue formation is stimulated by gonadal steroid hormones, including estrogens and androgens [8]. Patients with estrogen deficiency often experience bone loss [9], and patients with postmenopausal osteoporosis show reduced bone mineral density [10]. Androgen-deficient patients suffer from reduced bone mass [11, 12]. These results suggest that sex hormones have an important role in osteogenic differentiation and bone tissue metabolism.

Chlormadinone acetate (CMA) is a derivative of naturally secreted progesterone, one of the fundamental female hormones. CMA has been used as an oral contraceptive in hormone replacement therapy, and in combination with estrogen in contraception [13]. CMA shows high contraceptive efficacy as it inhibits ovulation through suppression of endogenous gonadotropin secretion and follicular growth and maturation [14]. A recent study reported that CMA promotes osteogenic differentiation and calcium deposition in human bone marrow-derived mesenchymal stem cells (hBMSCs) [15].

To date, there has been no study examining the effects of CMA on the odontogenic differentiation of hDPCs. Thus, the aim of this study was to investigate the effects of CMA on differentiation and mineralization of hDPCs and the role of extracellular signal-regulated kinase (ERK) as a mediator of CMA-stimulated odontogenic differentiation in hDPCs. The null hypothesis was that CMA has no effect on the odontogenic differentiation and mineralization of hDPCs.

## Methods

### Cell isolation and culture

Freshly extracted third molars from healthy patients were obtained from the Department of Oral and Maxillofacial Surgery, Chonnam National University Dental Hospital (Gwangju, Republic of Korea). Teeth were split under sterile conditions and pulp tissue was minced and plated in a 100-mm culture plate (Nunc, Roskilde, Denmark). Cells were cultured in α-minimum essential medium (α-MEM; Gibco) supplemented with 10% fetal bovine serum (FBS; Gibco, Invitrogen), 100 U/mL penicillin and 100 mg/mL streptomycin (Gibco, Invitrogen) in a humidified atmosphere of 5% CO2 at 37 °C. Cells passaged 3 to 5 times were used in this study. For mineralization experiments, cells were cultured in odontogenic induction media (OIM) with 50 μg/mL ascorbic acid (Sigma-Aldrich, St Louis, MO, USA) and 10 mmol/L β-glycerophosphate (Santa Cruz Biotechnology, Inc., Dallas, TX, USA).

### Cell viability assay

Cells were seeded in 96-well culture plates at a density of 1 × 10^4^ cells per well. Then, the cells were exposed to 0, 0.01, 0.1, 1, or 10 μM CMA (Sigma-Aldrich) for 48 h. Cell viability was examined using an EZ-Cytox cell viability assay kit (Daeil Lab Service, Seoul, Korea) according to the manufacturer’s recommendations. Briefly, 10 μL Ez-Cytox (tetrazolium salt) was added to the medium, and the cells were incubated at 37 °C for 3 h. Absorbance was measured at 420 nm using a spectrophotometer (VERSAmax multiplate reader; Molecular Devices, Sunnyvale, CA, USA).

### Quantitative real-time polymerase chain reaction (PCR)

Cells were seeded in 6-well culture plates at a density of 2 × 10^5^ cells per well. The cells were exposed to differentiation medium containing 0, 0.1, 1, or 10 μM CMA for 2, 5 and 7 days. Total RNA was isolated with the TRIzol reagent (Gibco, Invitrogen) according to the manufacturer’s instructions. cDNA was synthesized using the Maxime RT PreMix Kit (iNtRON Biotech, Seongnam, Korea). Quantitative real-time PCR was conducted using the QuantiTect SYBR Green PCR Kit (Qiagen, Valencia, CA, USA) in triplicate in a Rotor- Gene 6000 (Corbett Research, Sydney, Australia). The primer sequences are detailed in Table 1. All quantified values were normalized to endogenous β-actin. The data for gene expression were analyzed by the ΔΔCt method as described previously [16].

**Table 1:** Primer sequence used for real-time PCR in this study

Gene	Sequences(5’-3’)
ALP	(F) 5’-GGACCATTCCCACGTCTTCAC-3’,
	(R) 5’-CCTTGTAGCCAGGCCCATTG-3’;
OCN	(F) 5’-CATGAGAGCCCTCACA-3’
	(R) 5’-AGAGCGACACCCTAGAG-3’;
DSPP	(F) 5’-CAACCATAGAGAAAGCAAACCGC-3’
	(R) 5’-TTTCTGTTGCCACTGCTGGGAC-3’;
DMP-1	(F) 5’-ATGCCTATCACAACAAACG-3’
	(R) 5’-CTCCTTTATGTGACAACTGC-3’
β-actin	(F) 5’-GTGGGGCGCCCCAGGCACCA-3’
	(R) 5’-CTCCTTAATGTCACGCACGAT-3’.

### ALP staining

Cells were seeded in 24-well culture plates at a density of 2 × 10^4^ cells per well with differentiation medium containing 0, 0.1, 1, and 10 μM CMA with or without pretreatment with ERK inhibitor (U0126) for 7 days. Cultured hDPCs in OIM were used as a positive control. After 7 days, the samples were washed with PBS and fixed with 70% ice-cold ethanol, rinsed 3 times with deionized water, and then treated with 300 μL of ALP staining solution (1-Step NBT/BCIP Solution; Thermo Fisher Scientific Inc, Rockford, IL, USA) for 15 min. ALP staining was photographed using an Officejet pro L7580 scanner (HP, Palo Alto, CA, USA). For quantitative analysis, the stains were extracted with 10% (w/v) cetylpyridium chloride in 10 mmol/L sodium phosphate (pH = 7.0) for 15 min. ALP staining was quantified by measuring absorbance at 540 nm using a spectrophotometer (VERSAmax Multiplate Reader).

### Alizarin red staining

For alizarin red staining, hDPCs were cultured as mentioned with ALP staining. After 14 days, hDPCs were stained with 2% alizarin red stain solution (LIFELINE Cell Tech, Frederick, MD, USA) for 20 min and washed five times with sterile water. Alizarin red staining was photographed and staining density was quantified using an image analyzing program (Image J; National Institutes of Health, Bethesda, MD, USA).

### Western blot analysis

After hDPCs were exposured to OIM containing 1 μM CMA, cell lysates were prepared by solubilizing the cells with 1 mL of PBS-TDS (PBS, 1% Triton X-100, 0.05% sodium deoxycholate, 0.01% SDS, 0.5 μg/ml leupeptin, 1 mM EDTA, 1 μg/ml pepstatin, 0.2 mM PMSF) for 15 min on ice. The lysates were centrifuged at 12,000 rpm for 10 min to remove cell membranes, and protein concentrations were determined with a BCA assay kit (Sigma-Aldrich) using bovine serum albumin (BSA) as a reference point. Samples containing equal amounts of protein were separated by sodium dodecyl sulfate–polyacrylamide gel electrophoresis (SDS-PAGE) and transferred to nitrocellulose transfer membranes (Bio-Rad, Hercules, USA) for 2 h at 100 V. The membranes were blocked with 5% non-fat dry milk in PBS-T (PBS, 0.1% Tween 20) for 1 h at room temperature. Then, the membranes were incubated with anti-ERK and anti-phospho-ERK (Cell signaling, Denver, MA, USA) for 1 h with the primary antibodies, which were diluted 1:1000 in PBS. Then, horseradish peroxidase (HRP)-conjugated secondary antibodies, anti-mouse IgG or anti-rabbit IgG (Sigma-Aldrich), were used at a 1:5000 dilution for 1 h at room temperature. After three washes, chemiluminescent HRP (Millipore Corporation, Billerica, MA, USA) was applied for 30 to 60 s, and luminescence was detected with a Chemiluminescence Imaging System (Ez-capture; Atto, Tokyo, Japan).

### Statistical analysis

Experiment was performed at least twice which consisted of triplicate independent test. One-way ANOVA was performed and Tukey’s test was used for post hoc analysis. The SPSS 18.0 software program was used for all analysis (SPSS, Chicago, IL, USA). Differences were considered significant at *p* < .05.

## Results

### Cytotoxicity of CMA

**Fig. 1:**
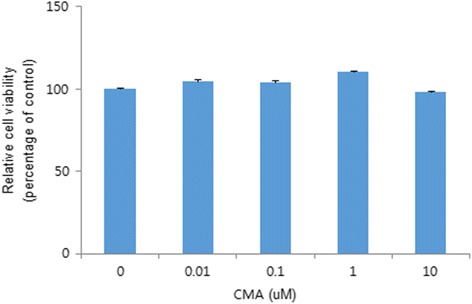
Effect of CMA on cell viability as measured by the WST-1 assay. Results are expressed as relative cell viability (percentage of control). There was no statistically significant difference between the groups (*p* > .05.)

To evaluate cellular viability following treatment with CMA, the WST-1 assay was performed (Fig. 1). Cell viability was not inhibited by CMA. There was no statistically significant difference between the untreated group and the CMA treated groups (*p* > .05).

### Effects of CMA on odontogenic differentiation

**Fig. 2:**
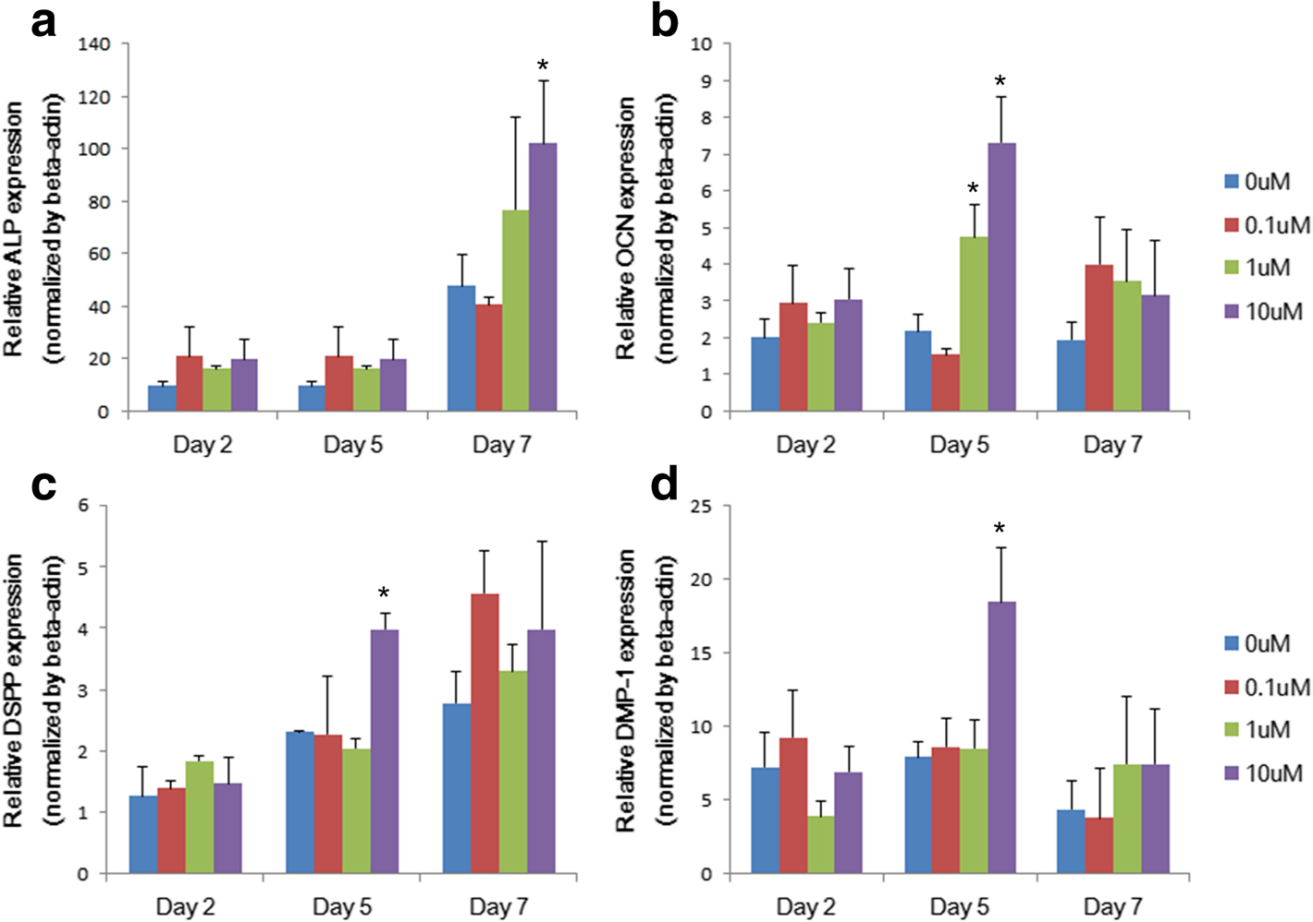
Expression profiles of ALP, OCN, DSPP, and DMP-1 during odontogenic differentiation by CMA in hDPCs, as measured by quantitative real-time PCR. The relative expression levels of **a** ALP, **b** OCN, **c** DSPP, and **d** DMP-1 genes were normalized to that of a housekeeping gene (β-actin). *Significant differences compared with the control (*p* < .05)

To investigate the effect of CMA on odontogenic differentiation in hDPCs, we assessed the levels of mRNA expression of odontogenic marker genes by quantitative real-time PCR (Fig. 2). The mRNA levels of the ALP gene increased significantly after 7 days of 10 μM CMA treatment. OCN mRNA levels increased significantly after treatment with 1 and 10 μM CMA at 5 days. DSPP and DMP-1 gene expression increased significantly with treatment of 10 μM CMA for 5 days (*p* < .05).

### Effects of CMA on mineralization

**Fig. 3:**
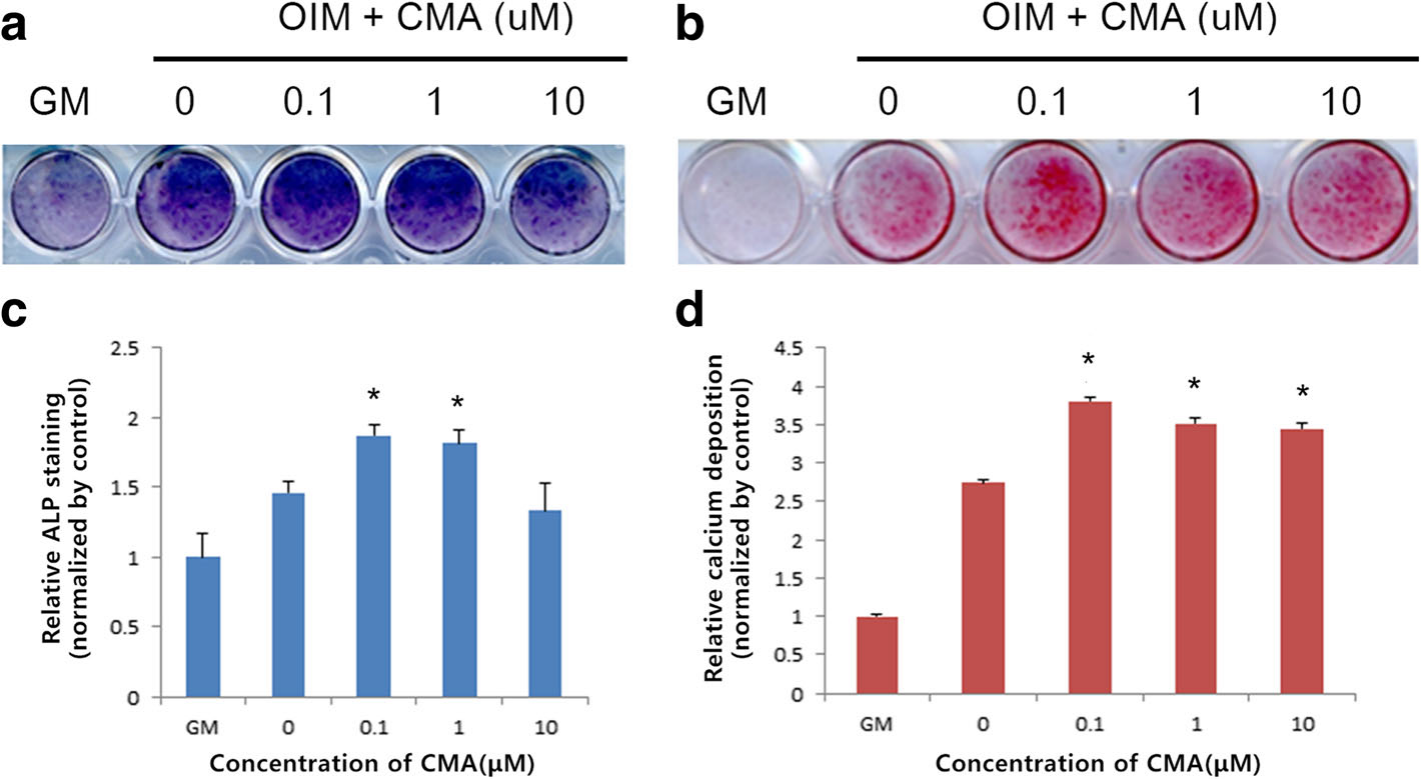
ALP activity evaluated by ALP staining and calcium nodule deposition evaluated by Alizarin red staining during odontogenic differentiation by CMA in hDPCs. **a** CMA increased ALP activity at concentrations of 0.1 and 1 uM. **b** CMA increased mineralized nodule formation at concentrations of 0.1, 1, and 10 uM. Quantification of ALP (**c**) and Alizarin red staining (**d**). OIM, odontogenic induction media.*Significant differences compared with cells treated with OIM alone (*p* < .05)

To investigate whether CMA regulates mineralization in hDPCs, we examined ALP activity by ALP staining and calcium nodule deposition by Alizarin red staining (Fig. 3). CMA up-regulated ALP activity at concentrations of 0.1 and 1 μM (Fig. 3a), and increased matrix mineralization at 0.1, 1, and 10 μM (Fig. 3b). These values were significantly different when compared with OIM alone (*p* < .05).

### Effects of CMA on ERK signaling pathway in hDPCs

**Fig. 4:**
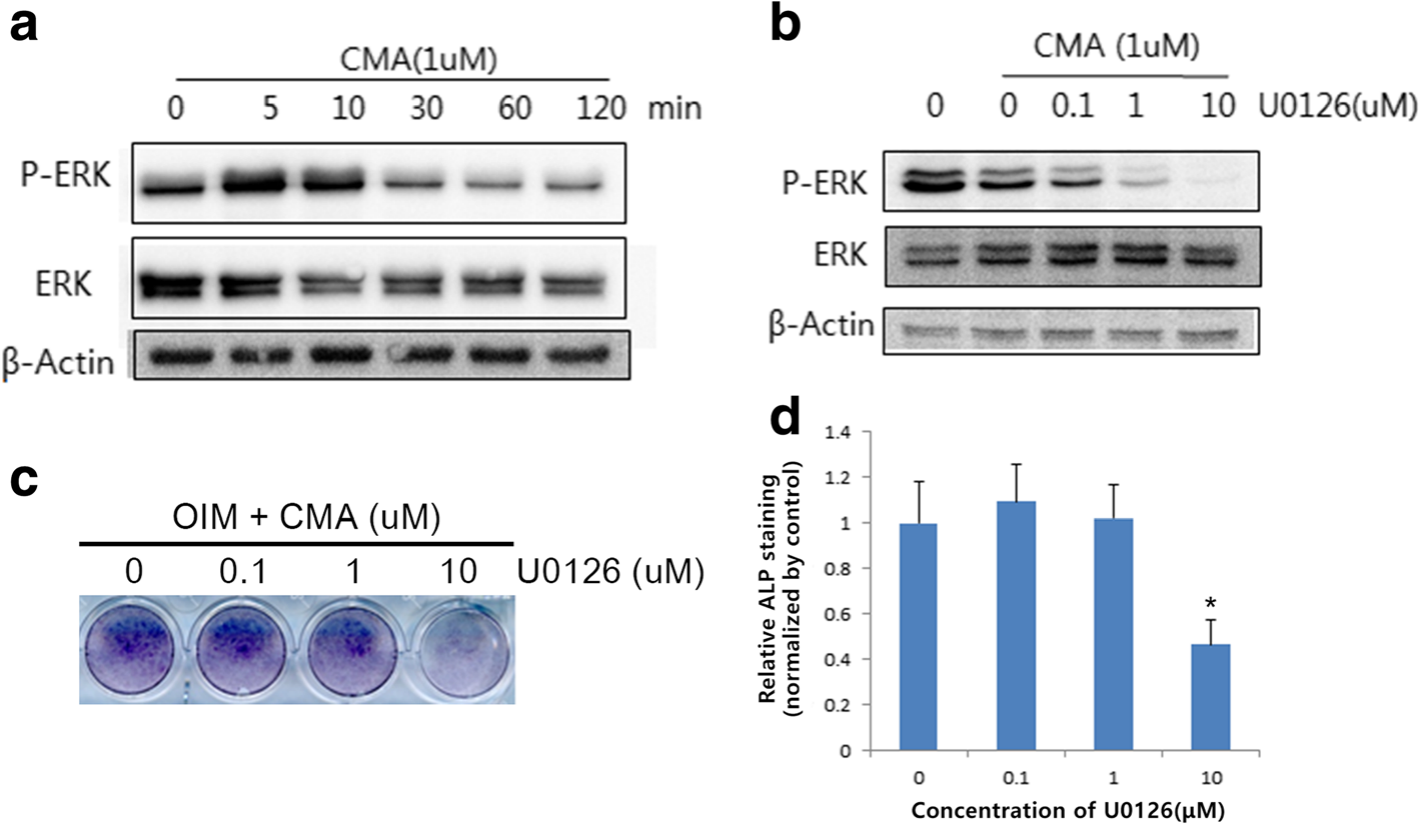
The signaling pathway of CMA-stimulated odontogenic differentiation in hDPCs, as determined by Western blot analysis and ALP staining. **a** Phosphorylation of ERK increased within 10 min and gradually decreased in a time-dependent manner. **b** ERK phosphorylation-dependent CMA expression was suppressed in a dose-dependent manner with U0126, an ERK inhibitor. **c** 10 uM U0126 significantly decreased CMA-induced odontogenesis. **d** Quantification of ALP staining by densitometry. *Significant differences compared with the control (*p* < .05)

To investigate the signaling pathways involved in CMA-mediated odontogenic differentiation of hDPCs, we examined the phosphorylation of ERK by Western blot. CMA treatment increased the phosphorylation of ERK within 10 min (Fig. 4a). U0126 is a selective ERK inhibitor. ERK phosphorylation following exposure to 1 μM CMA was inhibited by U0126 in dose-dependent manner (Fig. 4b). Furthermore, 10 μM U0126 significantly decreased CMA-induced ALP activity (Fig. 4c and d). These results suggest that CMA increases ERK phosphorylation.

## Discussion

CMA is a derivative of 17-hydroxyprogesternoe, which is chlorinated in position 6 and has an α-acetoxy-group at position 17. It was first synthesized in 1961 and has been used as an oral contraceptive. Previous studies suggested that CMA has strong progestogenic activity and moderate anti-androgenic properties [13]. However, no previous studies have investigated the effect of CMA on odontogenic differentiation of hDPCs. Only one report suggested that CMA promotes osteogenic differentiation and mineralization through stimulation of the differentiation of hBMSCs into mature osteoblasts through the ERK signaling pathway [15]. Because DPCs and BMSCs are very similar [17], we hypothesized that CMA plays a significant role in odontogenic differentiation in hDPCs.

In this study, ALP, OCN, DSPP, and DMP-1 were selected as specific odontoblast markers. ALP, which plays an important role in mineral deposition, is considered an early marker of osteo/odontogenic differentiation [18]. OCN, which regulates the mineral phase of bone and dentin, and DSPP, which is involved in the dentin mineralization process, are markers of late stages of osteo/odontogenic differentiation [19, 20]. DMP-1, which is predominantly expressed in odontoblasts, is required during the early and late stages of osteo/odontogenic differentiation [21]. In our study, we observed upregulation of odontogenic markers in the CMA-treated group after 5 or 7 days. Our results are consistent with those of previous reports involving hBMSCs [15].

We next investigated whether CMA regulates mineralization in hDPCs by ALP staining and Alizarin red staining. During early odontogenic differentiation, ALP can be used as a marker of specific proteins associated with pulp cells [22]. Alizarin red is an early-stage marker of matrix mineralization, which is a crucial step in the formation of calcified extracellular matrix in odontogenic differentiation [23]. Therefore, ALP activity and alizarin red staining can indirectly represent the mineralization capacity and differentiation ability of cells. In the present study, CMA-stimulated hDPCs showed increased ALP activity and formation of mineralized nodules. These results indicate that CMA is able to promote the mineralization and differentiation of hDPCs.

Mitogen-activated protein kinases (MAPKs) are serine/threonine kinases that are an essential component of many physiological processes, such as cell growth, proliferation, differentiation, and apoptosis [24]. The MAPK family is comprised of three actors, extracellular signal-regulated kinase (ERK), c-Jun N-terminal kinase (JNK), and p38 protein kinase [25]. We postulated that ERK may be involved in CMA-induced differentiation because the ERK pathway has been reported to contribute to odontogenic differentiation in hDPCs [26, 27]. Our results showed that CMA increased ERK phosphorylation within 10 min. In addition, a specific antagonist of ERK, U0126, inhibited CMA-induced phosphorylation of ERK and mineralization. These results suggest that the ERK pathway is a regulator of CMA-induced odontogenic differentiation in hDPCs.

As yet, there has been no study on the effects of progesterone, which is a component of CMA, on human dental pulp. Previous study only reported that the progesterone receptor is present in human dental pulp [28]. Progesterone plays an important role in the periodontium and bone. Progesterone alters periodontal ligament fibroblast metabolism and increases vascular permeability in periodontal tissue [29], and it activates bone metabolism and inhibits bone resorption [30]. Based on these results, we expected that progesterone would have an effect on dental pulp cells, including periodontium and bone. In this study, CMA was shown to promote odontogenic differentiation of hDPCs, as evidenced by the formation of mineralized nodules, the induction of ALP phenotypes, and upregulation of odontogenic markers. For the clinical usage, CMA should be used in local application because there might be some side effect when used in systemic application. The topical application of CMA on the exposed pulp tissue may be considerable. In this respect, if CMA is delivered to the pulp-dentin complex properly, CMA may provide a therapeutic effect for the regeneration of dental pulp tissues.

## Conclusion

Collectively, the current study demonstrated that CMA increased odontogenic differentiation markers and mineralization nodule formation, and these are regulated by the ERK signaling pathway. These results suggest that CMA may play a role in regeneration of pulp-dentin complex.

## Abbreviations

ALP: Alkaline phosphatase
CMA: Chlormadinone acetate
DMP: Dentin matrix protein
DSPP: Dentin sialophosphoprotein
ERK: Extracellular signal-regulated kinase
FBS: Fetal bovine serum
GM: Growth media
hBMSC: Human bone marrow-derived mesenchymal stem cells
hDPC: Human dental pulp cell
HRP: Horseradish peroxidase
IRB: Institutional review board
JNK: c-Jun N-terminal kinase
MAPK: Mitogen-activated protein kinases
MEM: Minimum essential medium
OCN: Osteocalcin
OIM: Odontogenic induction media
PBS: Phosphate-buffered saline
WST: Water-soluble tetrazolium
